# Cell Cycle Arrest in G_2_/M Phase Enhances Replication of Interferon-Sensitive Cytoplasmic RNA Viruses via Inhibition of Antiviral Gene Expression

**DOI:** 10.1128/JVI.01885-18

**Published:** 2019-02-05

**Authors:** Christian Bressy, Gaith N. Droby, Bryant D. Maldonado, Nury Steuerwald, Valery Z. Grdzelishvili

**Affiliations:** a Department of Biological Sciences, University of North Carolina at Charlotte, Charlotte, North Carolina, USA; b Levine Cancer Institute, Atrium Health, Charlotte, North Carolina, USA; University of Kentucky College of Medicine

**Keywords:** G_2_/M, Sendai virus, cell cycle, colchicine, mitotic inhibition of transcription, nonsegmented negative-strand RNA virus, paclitaxel, type I interferon, vesicular stomatitis virus

## Abstract

Vesicular stomatitis virus (VSV) (a rhabdovirus) and its variant VSV-ΔM51 are widely used model systems to study mechanisms of virus-host interactions. Here, we investigated how the cell cycle affects replication of these viruses using an array of cell lines with different levels of impairment of antiviral signaling and a panel of chemical compounds arresting the cell cycle at different phases. We observed that all compounds inducing cell cycle arrest in G_2_/M phase strongly enhanced the replication of VSV-ΔM51 in cells with functional antiviral signaling. G_2_/M arrest strongly inhibited type I and type III interferon (IFN) production as well as expression of IFN-stimulated genes in response to exogenously added IFN. Moreover, G_2_/M arrest enhanced the replication of Sendai virus (a paramyxovirus), which is also highly sensitive to the type I IFN response but did not stimulate the replication of a wild-type VSV that is more effective at evading antiviral responses. In contrast, the positive effect of G_2_/M arrest on virus replication was not observed in cells defective in IFN signaling. Altogether, our data show that replication of IFN-sensitive cytoplasmic viruses can be strongly stimulated during G_2_/M phase as a result of inhibition of antiviral gene expression, likely due to mitotic inhibition of transcription, a global repression of cellular transcription during G_2_/M phase. The G_2_/M phase thus could represent an “Achilles’ heel” of the infected cell, a phase when the cell is inadequately protected. This model could explain at least one of the reasons why many viruses have been shown to induce G_2_/M arrest.

**IMPORTANCE** Vesicular stomatitis virus (VSV) (a rhabdovirus) and its variant VSV-ΔM51 are widely used model systems to study mechanisms of virus-host interactions. Here, we investigated how the cell cycle affects replication of VSV and VSV-ΔM51. We show that G_2_/M cell cycle arrest strongly enhances the replication of VSV-ΔM51 (but not of wild-type VSV) and Sendai virus (a paramyxovirus) via inhibition of antiviral gene expression, likely due to mitotic inhibition of transcription, a global repression of cellular transcription during G_2_/M phase. Our data suggest that the G_2_/M phase could represent an “Achilles’ heel” of the infected cell, a phase when the cell is inadequately protected. This model could explain at least one of the reasons why many viruses have been shown to induce G_2_/M arrest, and it has important implications for oncolytic virotherapy, suggesting that frequent cell cycle progression in cancer cells could make them more permissive to viruses.

## Abstract (precis)

Vesicular stomatitis virus (VSV) (a rhabdovirus) and its variant VSV-ΔM51 are widely used model systems to study mechanisms of virus-host interactions. Here, we investigated how the cell cycle affects replication of VSV and VSV-ΔM51. We show that G_2_/M cell cycle arrest strongly enhances the replication of VSV-ΔM51 (but not of wild-type VSV) and Sendai virus (a paramyxovirus) via inhibition of antiviral gene expression, likely due to mitotic inhibition of transcription, a global repression of cellular transcription during G_2_/M phase. Our data suggest that the G_2_/M phase could represent an “Achilles’ heel” of the infected cell, a phase when the cell is inadequately protected. This model could explain at least one of the reasons why many viruses have been shown to induce G_2_/M arrest, and it has important implications for oncolytic virotherapy, suggesting that frequent cell cycle progression in cancer cells could make them more permissive to viruses.

## INTRODUCTION

Vesicular stomatitis virus (VSV) is a prototypic nonsegmented negative-strand (NNS) RNA virus (order *Mononegavirales*, family *Rhabdoviridae*). The 11-kb genome of VSV encodes five proteins that are all included in the enveloped, bullet-shaped VSV virion: nucleocapsid protein (N), phosphoprotein (P), matrix protein (M), glycoprotein (G), and large polymerase (L) (1). VSV is able to infect and replicate in a wide range of cell types (2). The pantropism of VSV is determined in part by the fact that several ubiquitously expressed cell surface molecules could be utilized by VSV for attachment to host cells, including the low-density lipoprotein receptor (LDLR) (3), phosphatidylserine (4–6), sialoglycolipids (7), and heparan sulfate (8). The ability of VSV to replicate in a wide range of cells is facilitated by the virus-encoded M protein, which helps VSV to evade innate antiviral responses in infected cells via inhibition of nuclear exit of host mRNAs, including transcripts for virus-induced antiviral genes (9–11).

VSV’s rapid replication, high virus yields in a wide range of cell types, and easily manipulated genome make it a popular model virus for studying basic mechanisms of virus-host interactions in NNS RNA and other cytoplasmic RNA viruses. However, although VSV is one of the best-studied viruses, the role of the cell cycle in VSV replication is still unclear, and previous studies that focused on different VSV recombinants and cell types have provided conflicting results. One study reported that the availability of certain translation initiation factors after successful G_0_-to-G_1_ cell cycle transition is crucial to sustain VSV replication in primary T lymphocytes (12). In contrast, a similar analysis of human hepatocellular carcinoma (HCC) cell lines showed that neither the G_0_-to-G_1_ transition nor the availability of translation initiation factors after the G_0_-to-G_1_ transition is essential for successful VSV replication in HCC cells (13). Another study showed that in the BHK-21 (baby hamster kidney fibroblast) cell line, the highest numbers of infectious particles were produced when cells were infected during the G_2_/M transition, although no mechanism was proposed to explain the observation (14).

Here, we examined the effects of the cell cycle on viral replication using VSV recombinants encoding either wild-type (WT) M or ΔM51 M protein and an array of human pancreatic ductal adenocarcinoma (PDAC) cell lines with different levels of impairment of type I interferon (IFN) signaling, which have been studied in detail in our previous studies (15–17). Most of our experiments in this study utilized VSV-ΔM51 and the Suit2 cell line. Compared to WT VSV, VSV-ΔM51 is sensitive to type I IFN antiviral responses, which allowed us to examine the effects of the cell cycle on cellular antiviral responses. VSV-ΔM51 has a deletion of methionine 51 in the VSV M protein, resulting in an inability of this protein to inhibit nucleus-to-cytoplasm transport of cellular mRNA, including antiviral transcripts (18–21). We chose Suit2 because it has limited permissiveness to VSV-ΔM51 due to functional type I IFN antiviral signaling (15, 22). As a result, the VSV-ΔM51/Suit2 combination is a useful model to study the effects of the cell cycle on replication of IFN-sensitive cytoplasmic viruses, as it allows one to detect decreases as well as increases of viral replication and examine changes in cellular antiviral responses in response to manipulations of the cell cycle.

Our data demonstrate that G_2_/M mitotic arrest strongly enhances the replication of VSV-ΔM51 (but not of WT VSV) and does so via inhibition of antiviral gene expression. A similar result was also observed for Sendai virus (SeV; a paramyxovirus), suggesting that the replication of at least some IFN-sensitive cytoplasmic RNA viruses can be strongly stimulated by this stage of the cell cycle. This model could explain at least one of the reasons why many viruses have been shown to induce G_2_/M arrest.

## RESULTS

### G_2_/M arrest strongly enhances replication of VSV-ΔM51.

**FIG 1:**
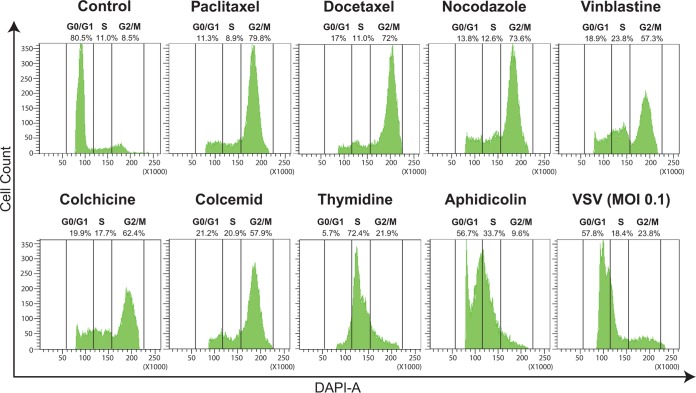
Induction of cell cycle arrest in Suit2 cells. Suit2 cells were treated (or mock treated [“Control”]) for 24 h by chemical compounds known to block the cell cycle in G_2_/M (500 nM paclitaxel, docetaxel, nocodazole, vinblastine, colchicine, or colcemid), in S (2 mM thymidine), or in G_1_/S (3 μM aphidicolin) phase. Alternatively, Suit2 cells were infected with VSV-ΔM51 at an MOI of 0.1 PFU/cell (the MOI was calculated based on virus titration on BHK-21 cells) for 24 h and then analyzed. Cell cycle stages were analyzed by flow cytometry with DAPI staining to determine nuclear DNA content, which was used to calculate the percentages of cells in different cell cycle phases. Single cells were gated via DAPI area and DAPI width signals and analyzed from a DAPI area histogram. Results show the data from one of three independent experiments.

To examine the role of the cell cycle in the permissiveness of cells to VSV-ΔM51, we used a panel of chemical compounds known to arrest the cell cycle at different phases (Fig. 1). Most of the compounds are commonly used to block the cell cycle in G_2_/M phase by either stabilization of microtubules (paclitaxel and docetaxel are microtubule-stabilizing agents [MSAs]) or destabilization of microtubules (nocodazole, vinblastine, colchicine, and colcemid are microtubule-destabilizing agents [MDAs]), which inhibit spindle dynamics, thereby leading to mitotic arrest. In addition, we included aphidicolin and thymidine to block the cell cycle in G_1_/S and S phases, respectively. First, we wanted to confirm that the chosen compounds arrested cells in the above-mentioned phases. Suit2 cells were treated for 24 h with the indicated compound and then stained with DAPI (4′,6-diamidino-2-phenylindole) and analyzed for cellular DNA content using flow cytometry (Fig. 1). As expected, the majority of mock-treated cells (“control”) (Fig. 1) were in G_0_/G_1_ phase. In contrast, paclitaxel, docetaxel, nocodazole, vinblastine, colchicine, and colcemid treatments dramatically shifted most of the cells to G_2_/M phase; thymidine treatment arrested most of the cells in S phase; and aphidicolin arrested most cells between G_1_ and S (Fig. 1). The flow cytometry data agreed with data from the confocal microscopy analysis demonstrating that only the compounds inducing G_2_/M arrest (as indicated by flow cytometry) induced easily visible chromatin condensation as well as the expected cell rounding that was observed using a phase-contrast microscope (data not shown). In contrast to the effects of these compounds on the cell cycle, infection of Suit2 cells with VSV-ΔM51 (indicated as “VSV” in Fig. 1) did not dramatically alter the cell cycle distribution, although a shift toward S and G_2_/M phases was observed (Fig. 1).

**FIG 2:**
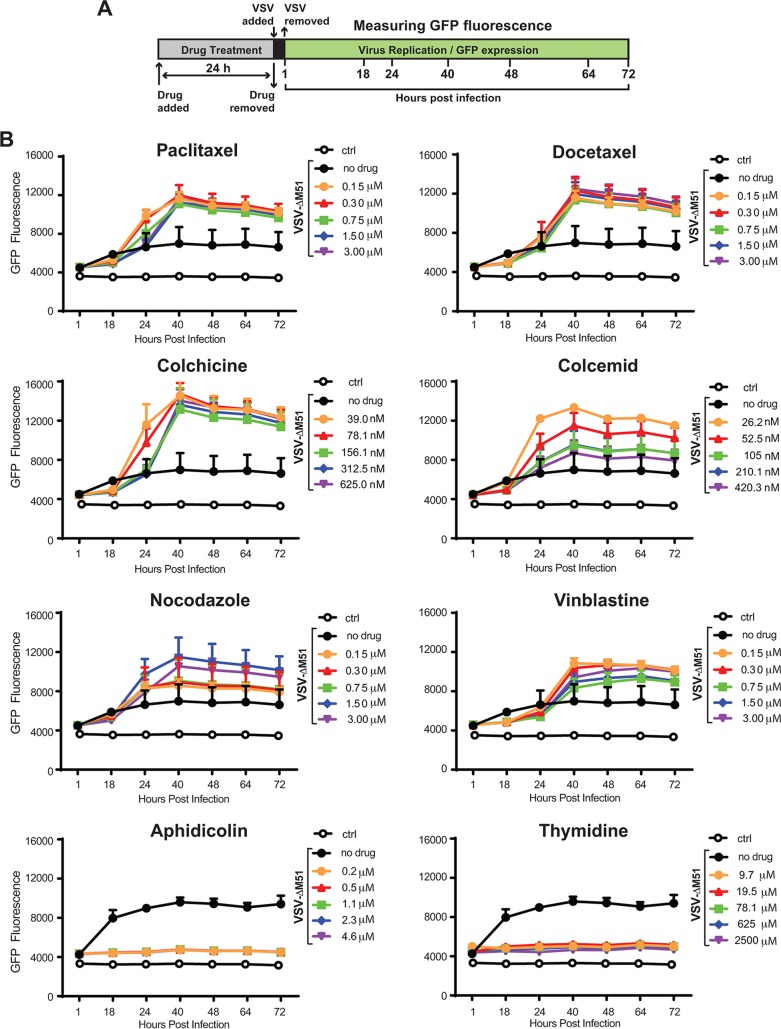
G_2_/M arrest strongly stimulates VSV-ΔM51 replication. (A) Experimental design scheme. (B) Suit2 cells were mock treated (control [“ctrl”]) or treated for 24 h with the indicated compounds at different concentrations and then infected with VSV-ΔM51 (indicated as “VSV”) at an MOI of 0.1 PFU/cell (the MOI was calculated based on virus titration on BHK-21). The level of GFP fluorescence was measured over the time from 1 h until 72 h p.i. The figure presents data representative of results from at least two independent experiments. The means and standard deviations (SD) of the means are indicated.

**FIG 3:**
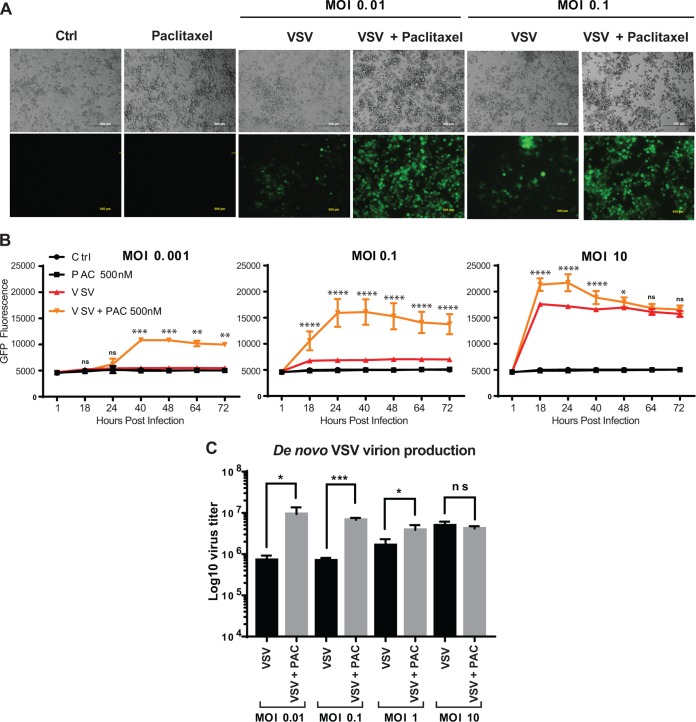
G_2_/M arrest stimulates VSV-ΔM51 replication under lower-MOI conditions. (A) Light and epifluorescence microscopy of Suit2 cells mock treated (Ctrl) or treated with paclitaxel (3 μM), VSV-ΔM51 (MOI of 0.01 or 0.1 PFU/ml [the MOI was calculated based on virus titration on BHK-21 cells]), or both for 72 h p.i. (B) Suit2 cells were seeded and washed with PBS before infection with 100 μl of VSV-ΔM51 at different MOIs (0.001, 0.1, or 10 PFU/cell [the MOI was calculated based on virus titration on BHK-21 cells]) for 1 h in medium without FBS. Cells were then washed and incubated for 72 h with 100 μl of medium (5% FBS) containing or not 500 nM paclitaxel. The measurements of GFP fluorescence were performed at the indicated time points. The data show results of one experiment representative of two, each performed in quadruplicates, and data represent the means and SD of the means. *, *P* < 0.05; **, *P* < 0.01; ***, *P* < 0.001; ****, *P* < 0.0001; ns, nonsignificant. The significance of the data was determined using two-way ANOVA with a Tukey posttest at a 95% confidence interval for comparison between VSV plus paclitaxel and VSV alone. (C) *De novo* virion production in the supernatant of Suit2 cells infected with VSV-ΔM51, incubated for 72 h, and treated or not treated with 3 μM paclitaxel (PAC). Virion production yield was measured by titrating the supernatants on BHK-21 cells using a standard plaque assay. The experiment was performed two independent times, and data are presented as the means and SD of the means. *, *P* < 0.05; ***, *P* < 0.001; ns, nonsignificant. The significance of the data was determined by using the two-tailed unpaired *t* test.

After confirming that chemical compounds were able to block the cell cycle in G_1_, G_1_/S, or G_2_/M phase, we examined the effect of the blocks on viral replication (Fig. 2). Suit2 cells were treated with different concentrations of each compound for 24 h and then infected with VSV-ΔM51 at a multiplicity of infection (MOI) of 0.1 PFU per cell (the MOIs here and elsewhere were calculated based on VSV-ΔM51 titration on BHK-21 cells, the reference cell line, which translates to a 20-fold-lower MOI in Suit2 cells). As VSV-ΔM51 has a green fluorescence protein (GFP) gene reporter between VSV genes G and L, we used GFP fluorescence to measure virus replication kinetics (Fig. 2A and B). As shown in previous studies, due to its downstream position between VSV genes G and L, virus-directed GFP expression can be used to measure virus replication levels, as it can be detected only if the virus genome is replicated (23). We observed that all chemical compounds blocking the cell cycle in G_2_/M caused a strong increase in VSV-ΔM51 replication (Fig. 2B). The strongest positive effect was observed for paclitaxel, docetaxel, and colchicine. In contrast, aphidicolin (G_1_/S-phase arrest) and thymidine (S-phase arrest) treatments strongly inhibited VSV-ΔM51 replication (Fig. 2B). While inhibition of virus replication by aphidicolin and thymidine could be attributed to negative effects of these treatments on cell physiology and/or specific inhibition of VSV replication, the observed strong stimulation of VSV-ΔM51 replication by every tested compound (MDA or MSA) inducing G_2_/M arrest was more intriguing. The observed increase in VSV-driven GFP expression (Fig. 3A and B) was also accompanied by an increase in *de novo* VSV virion production by paclitaxel-treated cells (Fig. 3C) (only paclitaxel was tested), confirming that paclitaxel-mediated G_2_/M arrest increased productive viral replication and not just VSV-driven GFP expression or stability. The increases in *de novo* virion production (Fig. 3C) and VSV-driven GFP expression (Fig. 3B) were particularly strong when cells were infected at a lower MOI. The effect of MOI on stimulation of viral replication by G_2_/M arrest is addressed again below in this study.

**FIG 4:**
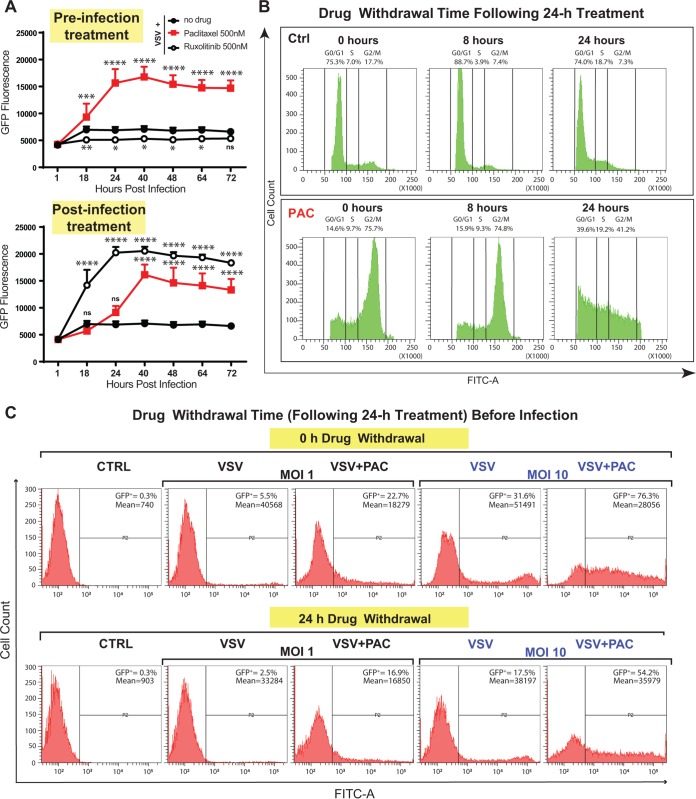
Paclitaxel is able to block the cell cycle in G_2_/M as well as improve viral replication even after its withdrawal from cells. (A) Suit2 cells were either treated with a compound (500 nM paclitaxel or 500 nM ruxolitinib) for 24 h before infection with VSV-ΔM51 (“preinfection treatment”) or first infected with VSV-ΔM51 and then treated with a compound (“postinfection treatment”). The level of GFP fluorescence was measured over the time from 1 until 72 h p.i. The data are representative of results from two independent experiments. *, *P* < 0.05; **, *P* < 0.01; ***, *P* < 0.001; ****, *P* < 0.0001; ns, nonsignificant. The significance of the data was determined using two-way ANOVA with a Tukey posttest at a 95% confidence interval for comparison of VSV plus paclitaxel or VSV plus ruxolitinib versus VSV alone. (B) Suit2 cells were treated with 500 nM paclitaxel (or mock treated) for 24 h and then monitored by a cell cycle analysis 0, 8, or 24 h after compound removal. Cell cycle stages were analyzed by flow cytometry with DAPI staining to determine nuclear DNA content, which was used to calculate the percentages of cells in different cell cycle phases. Single cells were gated via DAPI area and DAPI width signals and analyzed from a DAPI area histogram. (C) Suit2 cells either were treated with 500 nM paclitaxel (PAC) or remained untreated for 24 h. Paclitaxel (or medium) was then removed for 0 or 24 h before infection with VSV-ΔM51 (MOI of 1 or 10 PFU/cell [the MOI was calculated based on virus titration on BHK-21 cells]). After virus infection, incubation of cells continued for 12 h. The percentage of GFP-positive (GFP^+^) cells as well as the mean fluorescence were analyzed by flow cytometry and are indicated on the top right of each graph. The data represent results from at least two independent experiments.

Despite their various chemical structures and mechanisms of action (e.g., paclitaxel is an MSA, while colchicine is an MDA), every chemical compound arresting cells in G_2_/M phase also stimulates VSV-ΔM51 replication (Fig. 2), suggesting that G_2_/M arrest is required for the observed stimulation of VSV-ΔM51 replication. To further address this issue, we examined how different treatment schedules influence virus replication levels. Our previous studies showed that treatment with ruxolitinib (a JAK1/JAK2 inhibitor) enhances VSV-ΔM51 replication in cell lines with functional type I IFN signaling but only when ruxolitinib was present after infection (24). In agreement with that study, we observed a strong stimulation of VSV-ΔM51 replication in Suit2 cells that were infected and then treated with ruxolitinib, while no such effect was observed if cells were treated with ruxolitinib prior to infection (Fig. 4A). In contrast, paclitaxel treatment had a strong increase on VSV-ΔM51 replication in pre- or postinfection paclitaxel-treated Suit2 cells (Fig. 4A). If stimulation of VSV-ΔM51 replication by paclitaxel treatment prior to infection was due to mitotic arrest, we anticipated that the arrest would persist for at least some time after the removal of paclitaxel from the medium. To test this hypothesis, we treated Suit2 cells with or without paclitaxel for 24 h; removed paclitaxel; continued cell incubation without it for 0, 8, or 24 h; and then analyzed the cell cycle using flow cytometry. In agreement with our hypothesis, the majority of cells remained in G_2_/M phase 8 h after paclitaxel removal, and even 24 h after paclitaxel removal, a large portion of cells remained in G_2_/M phase (Fig. 4B). A similar result was observed for colchicine-treated cells (data not shown). Based on this result, we hypothesized that paclitaxel treatment would enhance VSV-ΔM51 replication even if paclitaxel-treated cells (24-h treatment) were incubated without it for another 24 h and then infected (“24-h drug withdrawal”) (Fig. 4C). In agreement with this hypothesis, paclitaxel increased VSV-ΔM51 replication under this experimental condition albeit to a lesser extent than under the “0-h drug withdrawal” condition (Fig. 4C). Interestingly, we consistently observed a lower number of GFP-positive cells in all groups when comparing 24-h to 0-h drug withdrawal conditions (Fig. 4C). We think that this may have happened because cells were allowed to grow an extra 24 h before virus infection, and it is possible that some changes (altered expression of cytokines or cell surface molecules or more dead cells, etc.) during those extra 24 h resulted in the decreased susceptibility and/or permissiveness of cells to VSV-ΔM51.

**FIG 5:**
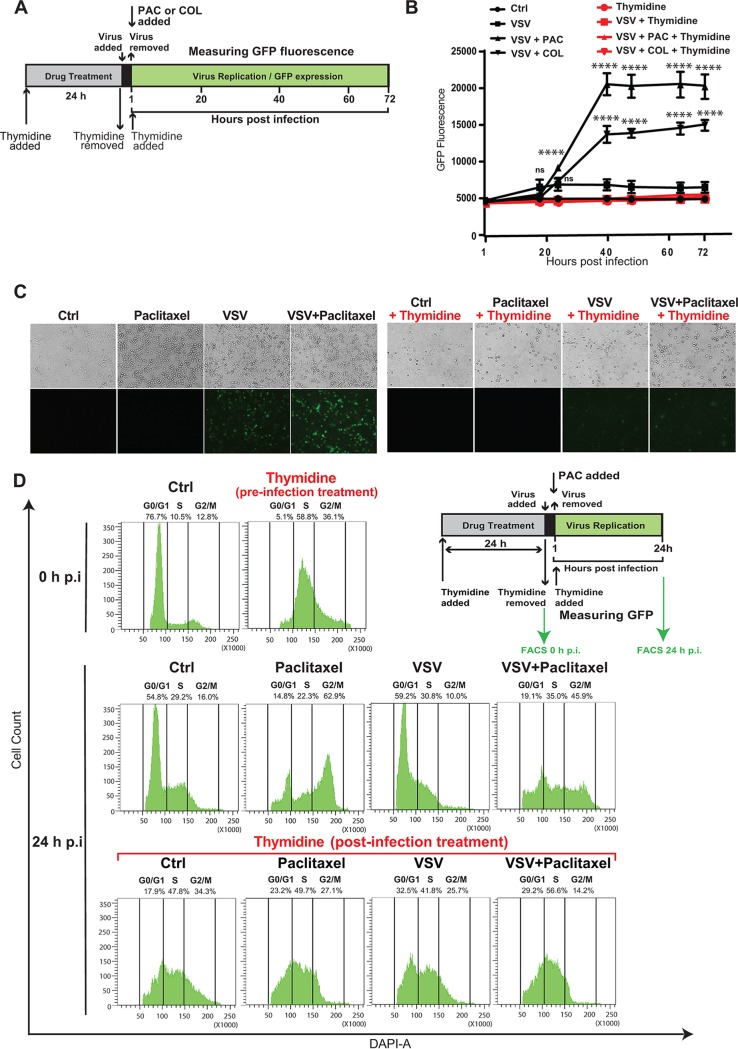
Treatment of Suit2 cells with thymidine impairs the ability of chemical compounds blocking the cell cycle in G_2_/M to improve VSV-ΔM51 replication. (A) Experimental design scheme. Suit2 cells were incubated with either 2 mM thymidine or medium with 10% FBS for 24 h. VSV-ΔM51 (0.1 PFU/cell) was used to infect cells for 1 h. After virus incubation, cells were washed with PBS. Medium containing either the vehicle, 500 nM paclitaxel (PAC), or 500 nM colchicine (COL) (plus 2 mM thymidine) was added for 72 h. (B) Kinetics of GFP expression by VSV over the time after the different treatments described above for panel A. The data are from two independent experiments, each performed in quadruplicates, and data represent the means and SD of the means (*, *P* < 0.05; ****, *P* < 0.0001; ns, nonsignificant). The significance of the data was determined using two-way ANOVA with a Tukey posttest at a 95% confidence interval for comparison of VSV plus paclitaxel or VSV plus colchicine versus VSV alone. (C) Suit2 cells were incubated with either 2 mM thymidine or the vehicle for 24 h. Cells were then infected (or mock infected) with VSV-ΔM51 (0.1 PFU/cell) for 1 h. After virus incubation, cells were washed, and medium containing either the vehicle, 500 nM paclitaxel, or 500 nM paclitaxel and 2 mM thymidine was added for 24 h. Light and epifluorescence microscopy of Suit2 cells were imaged. (D) Cell cycle stages were analyzed for cells described above for panel C using flow cytometry with DAPI staining to determine nuclear DNA content, which was used to calculate the percentages of cells in different cell cycle phases. Single cells were gated via DAPI area and DAPI width signals and analyzed from a DAPI area histogram. The data are representative of results from two independent experiments. FACS, fluorescence-activated cell sorter.

To further investigate whether G_2_/M arrest or the presence of drugs alone is required to increase VSV-ΔM51 replication, we blocked Suit2 cells in S phase with thymidine prior to and after VSV-ΔM51 infection with or without colchicine or paclitaxel (Fig. 5A). Our data showed that thymidine treatment suppressed stimulation of VSV-ΔM51 replication by colchicine (Fig. 5B) or paclitaxel (Fig. 5B and C), likely because it prevented the transition of S-phase-arrested cells to G_2_/M phase (Fig. 5D). Altogether, our data suggest that G_2_/M phase is required for the observed increase in VSV-ΔM51 replication by paclitaxel and other G_2_/M-arresting compounds.

### Stimulation of VSV-ΔM51 replication by G_2_/M arrest is due to inhibition of antiviral responses.

**FIG 6:**
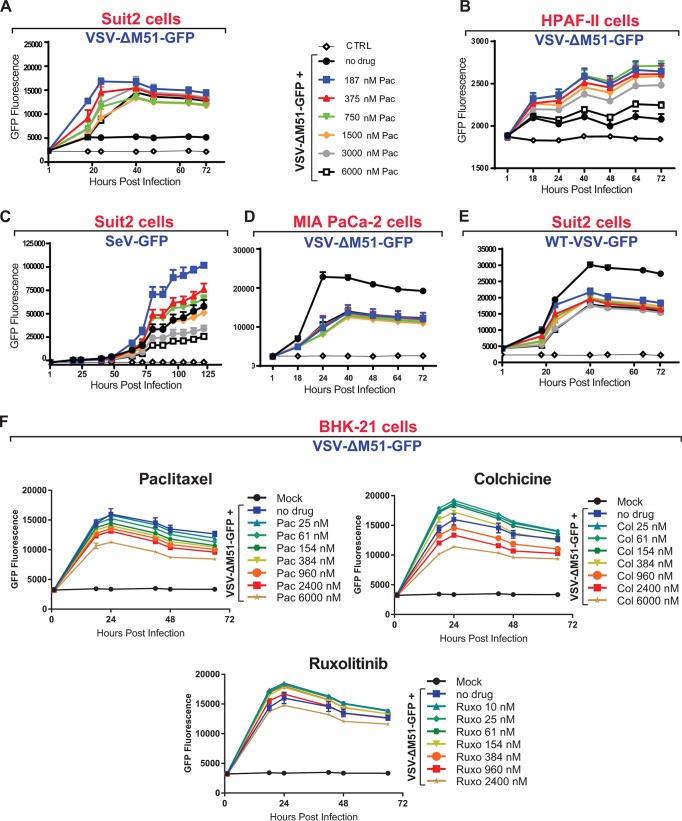
G_2_/M arrest improves viral replication only when active type I IFN signaling inhibits viral replication. (A to E) Suit2 cells (A, C, and E), HPAF-II cells (B), or MIA PaCa-2 cells (D) were treated with medium or different concentrations of paclitaxel (Pac) at the indicated concentration ranges for 24 h and then infected (or mock infected) with VSV-ΔM51 (MOI of 0.1 for Suit2 or MOI of 10 for HPAF-II cells), WT VSV (MOI of 0.1), or Sendai virus recombinant SeV-GFP (MOI of 0.1). The MOI for each virus was calculated based on virus titration on BHK-21 cells. The level of GFP intensity was measured in cells over time. (F) BHK-21 cells were treated 26 h prior to or following VSV-ΔM51 infection at an MOI of 0.01 with medium, paclitaxel, colchicine (Col), or ruxolitinib (Ruxo) at the indicated concentration ranges. After infection, virus replication was measured at regular intervals by way of GFP fluorescence. The means and SD of the means are indicated.

Two major mechanisms could explain the observed stimulation of VSV-ΔM51 replication by G_2_/M arrest. First, it is possible that in G_2_/M-arrested cells, some restriction factors of viral replication are inhibited. Alternatively, it is possible that during this cell cycle phase, some limiting host factors of VSV replication become available, thus enhancing viral replication. We decided to focus on the first hypothesis, which is consistent with our previous studies demonstrating that the ability of cells to mount functional type I IFN antiviral responses is a major determinant of permissiveness of cells to VSV-ΔM51 (15, 17, 24). To test this hypothesis, we analyzed the effect of paclitaxel on viral replication using different cell lines and viruses with various abilities to mount and evade type I IFN responses, respectively (Fig. 6). First, paclitaxel treatment increased VSV-ΔM51 replication not only in Suit2 (Fig. 6A) but also in HPAF-II (Fig. 6B) and AsPC-1 (data not shown) cells, all of which can mount a functional type I IFN response against VSV-ΔM51 (15). Second, in Suit2 cells, paclitaxel treatment stimulated the replication of Sendai virus (a paramyxovirus), another cytoplasmic NNS RNA virus which is also sensitive to type I IFN responses (Fig. 6C) (25). Importantly, this result shows that G_2_/M arrest can stimulate the replication of other cytoplasmic RNA viruses, via general mechanisms that are not limited only to VSV-ΔM51. Third, we did not observe any positive effects of paclitaxel on VSV-ΔM51 replication in MIA PaCa-2 cells, which are defective in type I IFN signaling (Fig. 6D) (15–17). Fourth, we did not observe any positive effects of paclitaxel on replication of WT VSV, which is capable of inhibiting type I IFN responses (Fig. 6E). These results also suggest that G_2_/M arrest does not stimulate viral replication by increasing the availability of some limiting host factors of VSV replication. If that was the case, then we would expect to see increases in WT VSV replication in Suit2 cells and VSV-ΔM51 replication in MIA PaCa-2 cells as well. We also tested the effects of paclitaxel and colchicine on replication of VSV-ΔM51 in BHK-21 cells, which are the most commonly used cells for production of VSV and many other viruses. As shown in Fig. 6F, while VSV-ΔM51 replication decreased in a dose-dependent manner in paclitaxel-treated cells, colchicine at several lower tested concentrations (25 to 384 nM) had a modest positive effect on VSV-ΔM51 replication (Fig. 6F). Importantly, VSV-ΔM51 replication in BHK-21 cells was slightly stimulated not only by colchicine but also by ruxolitinib (a JAK1/JAK2 inhibitor) (Fig. 6F), indicating that BHK-21 cells are not completely defective in type I interferon signaling, which agrees with a previous report (26).

Interestingly, we observed negative effects of paclitaxel on replication of VSV-ΔM51 in MIA PaCa-2 (Fig. 6D) and BHK-21 (Fig. 6F) cells and on replication of WT VSV in Suit2 cells (Fig. 6E). It is likely that paclitaxel has some negative effect on viral replication in all virus/cell line combinations, but it is directly observable only when the antiviral response is effectively evaded by virus (WT VSV) or when it is not functional (MIA PaCa-2 cells) or weak (BHK-21 cells) due to cellular defects.

### G_2_/M arrest inhibits expression of ISGs and antiviral IFNs.

**FIG 7:**
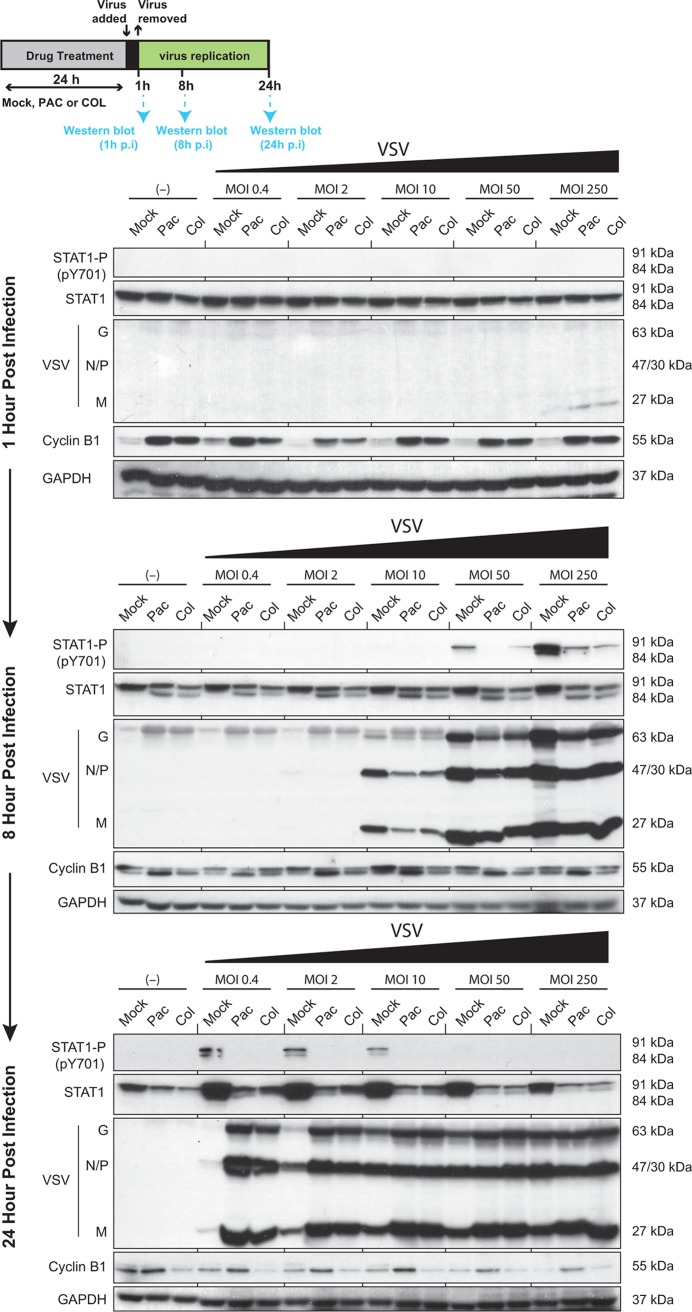
Inhibition of the type I interferon response in Suit2 cells blocked in G_2_/M allows an increase in viral replication. Suit2 cells were treated with 500 nM paclitaxel (Pac) or 500 nM colchicine (Col) or remained untreated for 24 h and then infected with VSV-ΔM51 at different MOIs (0, 0.4, 2, 10, 50, and 250 PFU/cell based on the titer determined on BHK-21 cells) for 1, 8, or 24 h. Western blotting shows the expression of phospho-STAT1 (STAT1-P) at Y701, STAT1, VSV proteins (G, N/P, and M), and cyclin B1. Protein names and protein sizes in kilodaltons are indicated on the left and right, respectively. GAPDH was used to confirm that protein loading was the same across the gel.

Type I IFN signaling is particularly protective against secondary infections of neighboring cells, which occur only under low-MOI infection conditions (when most cells are not infected during primary infection) and at later time points after infection (when virus spreads to neighboring cells) (27). Our data are consistent with this scenario, as the strongest positive effect of paclitaxel on VSV-ΔM51 replication was observed at lower tested MOIs (Fig. 3B and C). To measure VSV replication and type I IFN response levels at different MOIs and time points, Suit2 cells were treated with paclitaxel or colchicine for 24 h and infected with VSV-ΔM51 at different MOIs, and cellular lysates were then collected at 1, 8, and 24 h postinfection (p.i.) and analyzed for viral and cellular proteins using Western blot analysis. As expected, no viral proteins were detected at 1 h p.i. (Fig. 7). For this time point, we did not observe any changes in the total signal transducer and activator of transcription 1 (STAT1) level, and no STAT1 phosphorylation (indicative of type I IFN signaling activation) was detected. At the same time, strong accumulation of cyclin B was detected in paclitaxel- and colchicine-treated cells, which is expected for cells arrested in G_2_/M phase (Fig. 7). In agreement with our hypothesis that G_2_/M arrest stimulates VSV-ΔM51 replication via inhibition of type I IFN signaling, we did not observe any positive effect of paclitaxel or colchicine on VSV-ΔM51 replication at 8 h p.i., when VSV is still replicating mainly in initially infected cells. In fact, a clear inhibition of viral replication can be observed at this time point, which is likely due to some negative effects of these chemical compounds on the cellular environment for viral replication in the initially infected cells. This result also suggests that G_2_/M arrest does not stimulates viral replication by increasing the availability of some limiting host factors of viral replication. If paclitaxel or colchicine treatment would make a host factor more available to virus, then we would expect to see an increase in VSV-ΔM51 replication at an earlier time after infection and at a higher MOI.

Interestingly, despite this negative effect on viral replication at 8 h p.i., decreases in total STAT1 accumulation (under all conditions, even in mock-infected cells) as well as STAT1 phosphorylation (MOI of 50 and MOI of 250) could be seen (likely as a result of a lower total STAT1 level). At 24 h p.i., a clear positive effect of paclitaxel or colchicine on VSV-ΔM51 replication was observed but primarily when cells were infected at a lower MOI. Again, we observed a clear inhibition of total STAT1 accumulation (especially in virus-infected cells) as well as STAT1 phosphorylation (under all MOI conditions) (Fig. 7).

**FIG 8:**
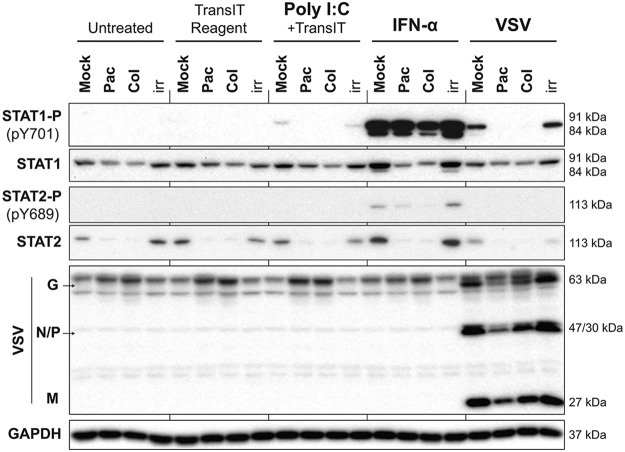
Induction of type I IFN signaling by viral and nonviral stimuli is inhibited in G_2_/M-arrested cells. Suit2 cells were treated for 25 h with the vehicle, paclitaxel, colchicine, or ruxolitinib at 500 nM. Cells were then treated with the vehicle (untreated), TransIT reagent (0.5%, vol/vol), poly(I:C) at 10 μg/ml plus TransIT reagent, IFN-α at 5,000 U/ml, or VSV at an MOI of 30 based on titration on BHK-21 cells. VSV was aspirated 1 h later, and medium was added to infected wells. Cells remained in treatment for a total of 4 h, after which total protein was isolated. Western blot results for STAT1 and -2 proteins and their phosphorylated forms are shown in addition to VSV proteins. GAPDH was used to confirm that protein loading was the same across the gel. Protein names and protein sizes in kilodaltons are indicated on the left and right, respectively.

To examine if the observed inhibition of total STAT1 accumulation (and phosphorylation) during G_2_/M arrest also happens in response to nonviral stimuli, we utilized IFN-α and poly(I:C), a mimic of viral double-stranded RNA and a potent inducer of type I IFN signaling. Suit2 cells were treated with the vehicle, paclitaxel, or colchicine at 500 nM for 25 h and then either treated with the vehicle (negative control), TransIT-TKO (TransIT) transfection reagent (control for the transfection reagent), poly(I:C) mixed with TransIT, or IFN-α or infected with VSV-ΔM51 (MOI of 30). Total protein was isolated at 4 h posttreatment and analyzed by Western blotting. The highest levels of phosphorylated STAT1 (STAT1-P) were found in mock-treated (no paclitaxel or colchicine) samples when cells were stimulated by IFN-α, while the lowest levels were found after poly(I:C) stimulation. Importantly, treatment of cells with paclitaxel or colchicine decreased STAT1-P levels induced by either IFN-α, poly(I:C), or VSV (Fig. 8). The paclitaxel-mediated inhibition of STAT1-P levels induced by IFN-α is not evident in Fig. 8 but was more apparent with a shorter exposure of this Western blot (data not shown). Again, as in Fig. 7, we observed a decrease in total STAT1 levels in cells treated with paclitaxel or colchicine, which likely determined lower levels of STAT1-P in those samples. A similar trend was observed for STAT2-P and STAT2, although STAT2-P levels were below our detection levels for cells induced with poly(I:C) or VSV. Not surprisingly for such an early stage in virus infection (4 h p.i.), no increase in VSV protein accumulation was observed for paclitaxel- or colchicine-treated cells (Fig. 8), which is consistent with our data for 8 h p.i. (Fig. 7).

**FIG 9:**
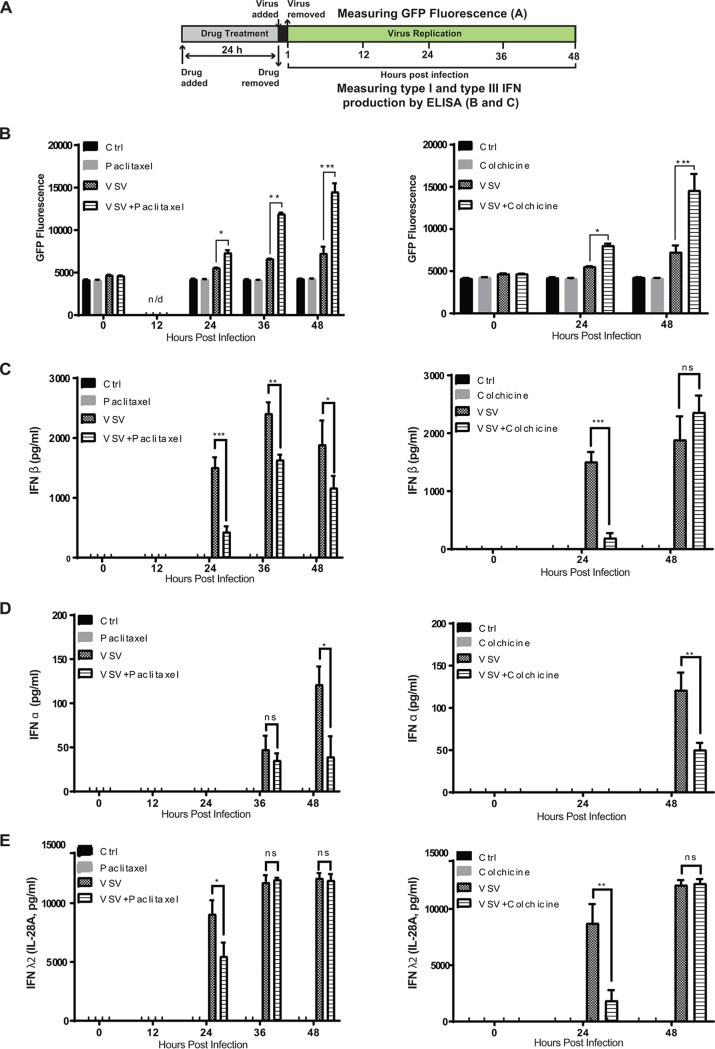
G_2_/M arrest inhibits expression of antiviral interferons. (A) Experimental design scheme. (B) Suit2 cells were treated (or mock treated) for 24 h with the compound (500 nM paclitaxel or colchicine) and then infected with VSV-ΔM51 (MOI of 0.1 PFU/cell [the MOI was calculated based on virus titration on BHK-21 cells]) for 48 h. The level of GFP intensity was measured in cells at different time points following viral infection. (C to E) In parallel, the production of IFN-α (C), IFN-β (D), and IFN-λ2 (IL-28A) (E) was quantified by an ELISA of the culture supernatants. The data represent the means and SD of the means. *, *P* < 0.05; **, *P* < 0.01; ***, *P* < 0.001; ns, nonsignificant. The significance of the data was determined by using the two-tailed unpaired *t* test at a 95% confidence interval.

**FIG 10:**
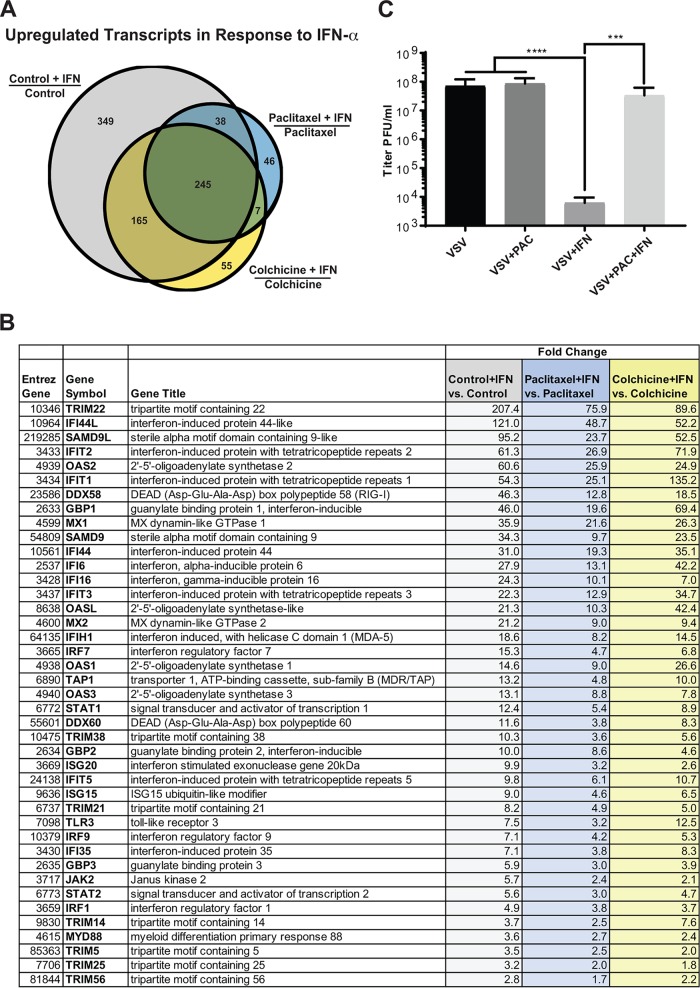
G_2_/M arrest inhibits upregulation of ISG expression in response to exogenously added IFN-α. (A) Suit2 cells were mock treated or treated for 24 h with 500 nM paclitaxel or 500 nM colchicine and then stimulated with 5,000 U/ml IFN-α for 4 h. Three biological repeats were conducted on 3 different days under each condition for RNA microarray analysis. Three comparisons were done (control plus IFN, paclitaxel plus IFN, and colchicine plus IFN versus control, paclitaxel, and colchicine, respectively). The number of transcripts upregulated in response to IFN-α treatment is indicated for each comparison in the Venn diagram. (B) List of the commonly upregulated transcripts in all three comparisons. (C) Suit2 cells were treated (or mock treated) with 500 nM paclitaxel for 24 h with or without 5,000 U/ml IFN-α and then infected with serial dilutions of VSV-ΔM51 and incubated for 24 h to calculate virus infectivity under each condition. The data represent the means and SD of the means from three independent experiments. ***, *P* < 0.001; ****, *P* < 0.0001. The significance of the data was determined by using one-way ANOVA with a Tukey posttest at a 95% confidence interval for comparisons of VSV-ΔM51 versus VSV-ΔM51 plus IFN, VSV-ΔM51 plus paclitaxel versus VSV-ΔM51 plus IFN, and VSV-ΔM51 plus paclitaxel plus IFN versus VSV-ΔM51 plus IFN.

Altogether, our data suggest that G_2_/M arrest primarily enhances secondary infection and replication of VSV-ΔM51 by inhibiting the establishment of an “antiviral state” in uninfected neighboring cells. The observed stimulation of viral replication could be due to inhibition of expression of IFN genes in response to initial viral infection and/or expression of antiviral IFN-stimulated genes (ISGs) in response to IFNs secreted by infected cells. To test this, we conducted two experiments. First, we examined kinetics of production of antiviral IFNs in response to viral infection (Fig. 9). In the second experiment, we analyzed global expression of antiviral genes in G_2_/M-arrested cells in response to the same amounts of exogenously added type I IFN (Fig. 10).

To determine the effect of G_2_/M arrest on antiviral IFN production, Suit2 cells were treated with paclitaxel or colchicine (or mock treated) for 24 h and then infected with VSV-ΔM51 for 1 h at an MOI of 0.1 (or mock infected) (Fig. 9A). Following infection, we examined virus replication-driven GFP expression (Fig. 9B), collected cell supernatants at different time points, and measured the production of the three most important antiviral IFNs, IFN-α (type I IFN), IFN-β (type I IFN), as well as IFN-λ2 (also known as interleukin-28A [IL-28A]), a type III IFN. The triggers for expression of type III IFNs and their activities are very similar to those of type I IFNs, but type I and III IFNs bind to unrelated heterodimeric receptors (28). Paclitaxel treatment (Fig. 9, left) resulted in not only statistically significantly increased viral replication at 24 and 48 h p.i. (Fig. 9B) but also decreased production of IFN-β at 24, 36, and 48 h p.i. (Fig. 9C; IFN-α at 48 h p.i. (Fig. 9D); and IFN-λ2 at 24 h p.i. (Fig. 9E). In general, similar results were obtained for colchicine-treated cells (Fig. 9, right), which were analyzed only at 0, 24, and 48 h p.i. (Fig. 9B to E). Colchicine treatment resulted in not only statistically significantly increased viral replication at 24 and 48 h p.i. (Fig. 9B) but also decreased production of IFN-β at 24 h p.i. (Fig. 9C), IFN-α at 48 h p.i. (Fig. 9D), and IFN-λ2 at 24 h p.i. (Fig. 9E). In general, these data demonstrate a clear negative correlation between stimulation of VSV-ΔM51 replication and inhibition of IFN production in cells treated with paclitaxel or colchicine. It is important to note that paclitaxel and colchicine treatments do not completely inhibit antiviral signaling including the production of IFNs, which are still strongly upregulated at many time points, for example, IFN-β at 48 h p.i. in colchicine-treated cells) but rather dampen antiviral responses at the time points (such as at 24 h p.i.) critically important for viral replication (Fig. 9).

Although the decreased production of antiviral IFNs would decrease ISG production and thus alone could explain the stimulation of viral replication, we wanted to examine if, in addition to inhibition of IFN production, G_2_/M arrest also independently inhibits transcription of antiviral ISGs in response to IFNs. To focus on this mechanism and nullify G_2_/M arrest-mediated stimulation of viral replication as well as inhibition of IFN production (both would modulate ISG expression), we treated Suit2 cells with paclitaxel or colchicine (or mock treated them) for 24 h and then treated cells with the same amounts of exogenously added IFN-α for 4 h, and total cellular RNA was isolated and analyzed by microarray analysis for the effects of paclitaxel and colchicine on ISG expression in response to IFN-α treatment. To address this question, we compared cells treated with no drug (“control”), paclitaxel, or colchicine and then exposed to IFN-α to cells treated with the same compound but not treated with IFN-α (“Control + IFN versus Control,” “Paclitaxel + IFN versus Paclitaxel,” and “Colchicine + IFN versus Colchicine” in Fig. 10 and Tables S1 to S4 in the supplemental material). We focused our analysis on the number of upregulated cellular transcripts (Fig. 10A and Tables S1 to S4) as well as their expression fold change (Fig. 10B and Tables S1 to S4). Both paclitaxel and colchicine treatments strongly decreased the number of transcripts upregulated in response to IFN-α treatment (Fig. 10A). Thus, IFN-α treatment upregulated the expression of 797 transcripts in control cells, 472 transcripts in colchicine-treated cells, and only 336 in paclitaxel-treated cells (Fig. 10A). Moreover, even for the 245 common transcripts upregulated in all cells treated with IFN-α, most of them were upregulated to a much lower level in paclitaxel- and colchicine-treated cells than in control cells. For example, for 2ʹ,5ʹ-oligoadenylate synthetase 2 (OAS2), IFN-α treatment resulted in a 60.6-fold change in control cells, a 24.9-fold change in colchicine-treated cells, and only a 25.9-fold change in paclitaxel-treated cells (Fig. 10B). Together, the microarray data demonstrate that G_2_/M arrest strongly inhibits IFN-mediated expression of antiviral genes even in response to the same amounts of IFN added to cells. To examine if G_2_/M arrest also functionally inhibits the response of cells to IFN treatment, Suit2 cells were treated with paclitaxel (or mock treated) for 24 h in the presence or absence of IFN-α treatment, and serial dilutions of VSV-ΔM51 were then added to cells to calculate viral yield (expressed as titer in PFU per milliliter) (Fig. 10C). We observed that IFN-α treatment dramatically reduced VSV-ΔM51 infectivity in control cells (7.1 × 10^7^ PFU/ml to 6.1 × 10^3^ PFU/ml); however, it was almost completely restored by paclitaxel treatment (to 3.3 × 10^7^ PFU/ml) (Fig. 10C). Therefore, paclitaxel treatment inhibited not only the expression of ISGs (Fig. 10A and B) but also the functional antiviral effects of IFN-α (Fig. 10C). Altogether, our data demonstrate that G_2_/M arrest inhibits the expression of antiviral IFNs as well as antiviral ISGs.

## DISCUSSION

In this study, we show that cell cycle arrest in G_2_/M phase can strongly enhance replication of VSV-ΔM51 via inhibition of antiviral gene expression. We observed this effect in all 3 tested human PDAC cell lines (Suit2, HPAF-II, and AsPC-1) that have functional type I IFN signaling (15–17). We also observed that paclitaxel treatment stimulated the replication of Sendai virus, another cytoplasmic NNS RNA virus (family *Paramyxoviridae*), which, like VSV-ΔM51, is highly sensitive to type I IFN responses. On the other hand, we did not observe any positive effects of G_2_/M arrest in the MIA PaCa-2 cell line, another human PDAC cell line, which, unlike Suit2, HPAF-II, and AsPC-1, has severely defective type I IFN antiviral signaling (15–17). Also, G_2_/M arrest of Suit2 cells did not stimulate replication of WT VSV, which is more effective in inhibiting type I IFN responses. We also showed that in cells with functional type I IFN signaling, G_2_/M arrest inhibited the expression levels of STAT1 and STAT2 and type I and III IFNs, as well as inhibiting the upregulation of ISGs in response to the same amounts of exogenously added type I IFN. Altogether, these and other data (e.g., G_2_/M arrest stimulated viral replication at later but not earlier time points after infection and under low- but not high-MOI conditions) suggest that G_2_/M arrest stimulates viral replication via inhibition of antiviral responses rather than by increasing the availability of some limiting host factors of viral replication. In the latter case, we would expect to see increased VSV-ΔM51 replication in MIA PaCa-2 cells and WT VSV replication in Suit2 cells.

Several previous reports agree with our data. One study showed that in BHK-21 cells, the highest numbers of infectious particles were produced when cells were infected during the G_2_/M transition, although no mechanism was proposed to explain that observation (14). In that study, BHK-21 cells were initially arrested at the G_1_/S phase with aphidicolin, the block was then released, and synchronously progressing cells were infected with an attenuated VSV-GFP recombinant at different time points after removal of the G_1_/S block (14). We also observed some improvement in VSV-ΔM51 replication in BHK-21 cells treated with colchicine. Importantly, VSV-ΔM51 replication in BHK-21 cells was slightly stimulated not only by colchicine but also by ruxolitinib (a JAK1/JAK2 inhibitor), indicating that BHK-21 cells are not completely defective in type I interferon signaling, which agrees with a previous report (26). In contrast to BHK-21 cells, no stimulation of VSV-ΔM51 replication was observed in MIA PaCa-2 cells treated with ruxolitinib, suggesting that they are more defective in type I IFN signaling (data not shown).

In another study, paclitaxel treatment stimulated the replication of Maraba virus, another member of the family *Rhabdoviridae*, genus *Vesiculovirus*, in breast cancer cell lines *in vitro* and *in vivo* (29). Interestingly, the effect was observed only in two of the three tested cell lines. Both EMT6 and 4T1 cells, in which sensitization to the virus was observed, produced IFN-β in response to Maraba virus infection, and IFN-β production was inhibited by paclitaxel treatment. In contrast to EMT6 and 4T1 cells, E0771 cells, which were refractory to this effect of paclitaxel, showed defective IFN-β production in response to Maraba virus infection (with or without paclitaxel treatment). In general, these data agree with results of our study demonstrating that, as G_2_/M arrest stimulates viral replication via inhibition of antiviral signaling, the effect can be seen only where this antiviral pathway is functional.

Another study showed that microtubule-destabilizing agents (MDAs), including colchicine and nocodazole, enhanced the replication of VSV-ΔM51 in several non-PDAC cancer cell lines (30). Although that study did not look at the effects of colchicine and nocodazole on the cell cycle in treated cells and did not test MSAs, it showed that these treatments inhibited ISG production following viral infection due to specific disruption of type I IFN mRNA translation (thus, ISG expression was inhibited due to the lower levels of IFN production by treated cells) (30). Importantly, in contrast to our results, in that study, colchicine treatment did not inhibit total STAT1 expression (or STAT1 phosphorylation) and did not overcome the antiviral effects of type I IFNs (30). Our data also show inhibition of type I (as well as type III) IFN production in cells treated with colchicine (MDA) or paclitaxel (MSA), which could be at least in part due to disruption of type I IFN mRNA translation, as shown previously (30). However, in our study, colchicine and paclitaxel overcame the antiviral effects of type I IFNs, and when cells were treated with the same amounts of exogenously added type I IFN as control cells, we observed a dramatic inhibition of ISG upregulation (the number of upregulated ISGs as well as the degree of their upregulation in G_2_/M-arrested cells), suggesting that, in addition to the reported colchicine-mediated inhibition of type I IFN mRNA translation (30), VSV-ΔM51 replication in G_2_/M-arrested cells is stimulated due to transcriptional repression of antiviral gene expression (IFNs and ISGs). Future studies will examine whether the discrepancies between our studies were due to differences between cell lines or our experimental procedures.

We think that such transcriptional repression is likely a consequence of a global repression of cellular transcription during the G_2_/M transition, so-called mitotic inhibition of transcription (31). The diminished transcription during mitotic arrest is well documented (32–38). Mitotic inhibition of transcription not only is a consequence of high condensation of chromatin during mitosis but also is achieved via multiple mechanisms, including mitosis-specific phosphorylation and displacement of the general transcription factor TFIID from the prophase nucleus to the mitotic cytoplasm around the time of nuclear envelope breakdown (34). Our data demonstrating the inhibition of antiviral gene expression during G_2_/M arrest agree with data from several previous studies. Thus, lower levels of IFN production were shown for mouse L cells (strain L-929) at late G_2_ phases of the cell cycle (39). Also, several studies reported inhibition of IFN production in various cell types after MSA treatment: in FS-4 human foreskin fibroblast cells by vinblastine or colchicine (40), in mouse bone marrow-derived macrophages and resident peritoneal macrophages by nocodazole (41), in mouse spleen cells by colchicine (42), and in a human lymphoblastoid cell line by colchicine (43). Also, MSA-mediated inhibition of IFN activities by colchicine and nocodazole was shown in 3T3-Swiss mouse fibroblasts (44). More recently, an unfavorable effect of colchicine in combination with IFN-α was reported for treated chronic hepatitis C patients (45). Although this epidemiological study did not examine the mechanism, the observation could be related to inhibition of IFN responses in colchicine-treated infected cells.

Our model suggests that even though replication of many viruses occurs exclusively in the cytoplasm, the cell cycle could affect the replication of such cytoplasmic viruses by modulating the ability of cellular transcriptional machinery to transcribe antiviral genes. It is possible that when cells are transitioning via G_2_/M phase, they are unable to adequately respond to viral assault by producing sufficient amounts of antiviral transcripts due to mitotic inhibition of transcription. This model could explain at least one of the reasons why many viruses (including DNA viruses, retroviruses, and RNA viruses [nuclear and cytoplasmic]) have been shown to induce G_2_/M arrest (46). For example, Borna disease virus (order *Mononegavirales*, family *Bornaviridae*) (replicates in the nucleus) nucleoprotein interacts with the CDC2-cyclin B1 complex and induces a delayed G_2_/M transition (47). Virus-mediated induction of G_2_/M arrest was also shown for other viruses, including JC polyomavirus (48), serotype 3 reoviruses (49), simian virus 40 (50), human parvovirus B19 (51), human papillomavirus 1 (52, 53), chicken anemia circovirus (54), herpes simplex virus 1 (55), herpes simplex virus 6 (56), hepatitis B virus (57), human immunodeficiency virus type 1 (58, 59), and Zika virus (60). We envision the G_2_/M phase as the “Achilles’ heel” of the infected cell, a phase during cell cycle progression when the cell is inadequately protected and thus is permissive to viral infection and replication. However, not all viruses need to induce G_2_/M cell cycle arrest or even benefit from it if they are well equipped to evade antiviral signaling. This could explain why WT VSV, which is able to evade innate antiviral responses in infected cells, did not benefit from G_2_/M arrest in our study and why VSV does not induce G_2_/M arrest.

Our data have interesting implications for oncolytic virotherapy, which utilizes attenuated VSV recombinants and other viruses that preferentially replicate in and kill cancer cells while leaving nonmalignant cells unharmed. Numerous preclinical studies demonstrated the effectiveness of VSV as an oncolytic virus (OV) (61–63), and a safe VSV recombinant, VSV-hIFNbeta-NIS (encodes human IFN-β and a sodium iodide symporter), is currently being tested in the United States in several phase I clinical trials against various malignancies (see details at ClinicalTrials.gov for trials under registration numbers NCT02923466, NCT03120624, and NCT03017820). We speculate that frequent cell cycle progression in cancer cells makes them more permissive to attenuated VSV recombinants and other OVs, and this mechanism could contribute to the oncoselectivity of viruses. This is an important issue as it is still unclear why cancer cells are generally more permissive to viruses (such as VSV-ΔM51) than nonmalignant cells. The oncoselectivity of attenuated VSV recombinants is mainly based on defective or reduced type I IFN responses in cancer cells (13, 19, 64–70). These responses are generally unfavorable for tumor formation and spread, as they are antiproliferative, antiangiogenic, and proapoptotic (71). Several mechanisms have been shown to downregulate or inactivate type I IFN responses in cancer cells, including IFN signaling inhibition by MEK/extracellular signal-regulated kinase (ERK) signaling (72) or epigenetic silencing of the IFN-responsive transcription factor interferon regulatory factor 7 (IRF7) or IRF5 (73). Here, we propose that continuous cell cycle transition, a hallmark of cancer cells, could be another factor of oncoselectivity for many viruses, also facilitating viral replication via inhibition of antiviral responses in dividing cancer cells.

## MATERIALS AND METHODS

### Viruses and cell lines.

The recombinant VSV-ΔM51 was described previously (74). It has a deletion of the methionine at amino acid position 51 of the matrix protein and the green fluorescent protein (GFP) open reading frame (ORF) inserted at position 5 of the viral genome (between the VSV G and L genes). WT VSV is similar to VSV-ΔM51 (and has the GFP ORF inserted at the same position) but has WT M (75). The recombinant Sendai virus SeV-GFP (SeV-GFP-F_mut_), which has been described previously (76), has the GFP ORF at position 1 of the viral genome and a mutation in the cleavage site of the fusion (F) protein, allowing F activation and production of infectious virus particles in cells without acetylated trypsin added to the medium. VSV-ΔM51 was grown on BHK-21 cells, and Sendai virus was grown on Vero (ATCC CCL81) cells. Viral titers for both viruses were determined by a standard plaque assay on BHK-21 cells and expressed as PFU per milliliter. The following human PDAC cell lines were used in this study: Suit2 (77), HPAF-II (ATCC CRL-1997), and MIA PaCa-2 (ATCC CRL-1420). The human origin of all these PDAC cell lines was confirmed by partial sequencing of KRAS and actin. As expected, all PDAC cell lines had a mutation in KRAS, as is typical for PDACs (17, 24). The BHK-21 baby hamster kidney fibroblast cell line (ATCC CCL-10) was used to grow viruses and determine their titers. MIA PaCa-2 and Suit2 cells were maintained in Dulbecco’s modified Eagle’s medium (DMEM) (catalog number 10-013-CV; Cellgro), while HPAF-II and BHK-21 cells were maintained in modified Eagle’s medium (MEM) (catalog number 10-010-CV; Cellgro). All cell growth media were supplemented with 10% fetal bovine serum (FBS; Gibco), 4 mM l-glutamine, 900 U/ml penicillin, 900 μg/ml streptomycin, and 1% nonessential amino acids. MEM was additionally supplemented with 0.3% (wt/vol) glucose. Cells were kept in a 5% CO_2_ atmosphere at 37°C. For all experiments, PDAC cell lines were passaged no more than 15 times.

### Chemical compounds.

The following compounds were used in this study: ruxolitinib (INCB018424, Jakafi/Jakavi, catalog number S1378; Selleck Chemicals), paclitaxel (catalog number S1150; Selleck Chemicals), docetaxel (catalog number S1148; Selleck Chemicals), colchicine (catalog number C9754; Sigma-Aldrich), colcemid (catalog number 10295892001; Roche), nocodazole (catalog number S2775; Selleck Chemicals), vinblastine (catalog number S4505; Selleck Chemicals), aphidicolin (catalog number A0781; Sigma-Aldrich), and thymidine (catalog number T9250; Sigma-Aldrich).

### Cell cycle analysis.

Suit2 cells (1.0 × 10^6^ cells) were seeded in 6-well plates in DMEM containing 10% FBS. When cells became confluent, they were washed with phosphate-buffered saline (PBS) and treated or not for 24 h with different chemical compounds (500 nM paclitaxel, docetaxel, colchicine, nocodazole, or vinblastine; 3 μM aphidicolin; or 2 mM thymidine) in DMEM with 5% FBS. Alternatively, Suit2 cells were infected by VSV-ΔM51 for 24 h and analyzed for the effect of viral replication on the cell cycle. For this, Suit2 cells were washed with PBS and then infected (or mock infected) with 500 μl of VSV-ΔM51 at an MOI of 0.1 PFU/cell for 1 h at 37°C in DMEM without FBS. Virus was then removed, and 1 ml of culture medium containing 5% FBS was added for 23 h. After the treatment, cells were washed with PBS, harvested, centrifuged 2 times at 1,200 rpm at 4°C for 5 min, and resuspended in 300 μl of sodium citrate buffer containing 0.1% Triton X-100, 100 μg/ml RNase, and 1 mg/ml DAPI. The cell cycle distribution was determined by flow cytometry analysis performed using a BD LSR Fortessa instrument (BD Biosciences). To analyze the cell cycle after paclitaxel withdrawal, Suit2 cells were treated with 500 nM paclitaxel for 24 h; the compound was then removed and replaced with DMEM (5% FBS) for 0, 8, or 24 h; and cell cycle analysis was conducted as described above.

### Analysis of GFP-positive cells by flow cytometry.

Suit2 cells (1.0 × 10^6^ cells) were seeded in 6-well plates in DMEM or RPMI medium containing 10% FBS. When cells became confluent, they were washed with PBS and infected or mock infected with 500 μl of VSV-ΔM51 at different MOIs (PFU per cell) for 1 h at 37°C in DMEM without FBS. The medium was then removed, and 1 ml of culture medium (5% FBS) containing 500 nM paclitaxel and/or 3 μM ruxolitinib was added. The percentage of GFP-positive cells and the mean fluorescence were analyzed by flow cytometry on a BD LSR Fortessa instrument (BD Biosciences) at 24 h p.i. using the fluorescein isothiocyanate area (FITC-A) channel. For some experiments, Suit2 cells were pretreated or not with 1 ml of paclitaxel in cell culture medium containing 5% FBS for 24 h, and paclitaxel was then removed for 0 or 24 h before infection with VSV-ΔM51. Next, cells were washed with PBS and infected or mock infected with 500 μl of VSV-ΔM51 at different MOIs for 1 h at 37°C in culture medium containing 0% FBS. Finally, the medium was removed, 1 ml of medium was added, and the analysis of cell fluorescence by flow cytometry was conducted at 12 h p.i.

### Effect of chemical compounds on virus replication and virion production.

Cells (5.0 × 10^4^ cells) were seeded in 96-well plates in corresponding culture medium containing 10% FBS. When cells became confluent, they were pretreated for 24 h with 100 μl of either medium (5% FBS) or medium with compounds at different concentrations, washed with PBS, and infected with 50 or 100 μl of virus (MOI of 0.1 PFU/cell for Suit2, MOI of 10 PFU/cell for HPAF-II, and MOI of 0.01 PFU/cell for MIA PaCa-2 cells [the MOI was calculated based on virus titration on BHK-21 cells]) for 1 h at 37°C in culture medium containing 0% FBS. Next, the medium was removed, and 100 μl of either medium (5% FBS) or medium with drug was added for 72 h. Alternatively, for some experiments, when cells reached confluence, they were washed with PBS and directly treated or not with 100 μl of virus at different MOIs for 1 h at 37°C in culture medium without FBS. The medium was then removed, and 100 μl of either medium (5% FBS) or medium with the compound was added for 72 h. Analysis of GFP fluorescence was performed at 1, 18, 24, 40, 48, 64, and 72 h p.i. with a CytoFluor series 4000 fluorescence multiwell plate reader (excitation filter of 485/20 nm, emission filter of 530/25 nm, and gain of 75; Applied Biosystems). For *de novo* virion production, supernatants of 96-well plates were collected at 3 days p.i., and plaque assays were performed on BHK-21 cells to measure virus yield.

### Poly(I:C) transfection.

Thirty minutes prior to transfection, poly(I:C) at a final concentration of 10 μg/ml was mixed with TransIT-TKO transfection reagent at 0.5% (vol/vol) (catalog number MIR 2154; Mirus). After treatment with chemical drugs, cells were washed once with 1 ml of PBS, and 1 ml of the transfection mixture was added to each well of a 24-well plate. The mixture was aspirated upon total protein isolation.

### RNA microarray analysis.

Three biological repeats were conducted on 3 different days under each condition for RNA microarray analysis. Suit2 cells (1.0 × 10^6^ cells) were seeded in 6-well plates in DMEM containing 10% FBS. When cells became confluent, they were washed with PBS and treated or mock-treated for 24 h with 500 nM paclitaxel or 500 nM colchicine in DMEM with 5% FBS. Cells were then washed with PBS and left untreated or treated with 1 ml of human recombinant IFN-α (catalog number 407-294; Millipore) for 4 h at 37°C in DMEM without FBS. Cellular RNA was extracted with TRIzol (Life Technologies) according to the manufacturer’s protocol, with slight modification. In brief, following the first phase of separation, the aqueous layer was transferred to a new tube. Next, 500 μl of TRIzol and 100 μl of chloroform were added, and phase separation was repeated. The integrity of the RNA was verified by an Agilent 2100 Bioanalyzer profile (Agilent Technologies Inc., Santa Clara, CA, USA). RNA integrity number (RIN) values were ≥7. Samples were reverse transcribed, amplified, and labeled using the 3ʹ IVT Express kit (Affymetrix). The resultant labeled cRNA was purified and fragmented according to the vendor’s instructions. The cRNA samples together with probe array controls were hybridized onto Affymetrix human genome U133^+^ PM array strips, which cover more than 47,000 transcripts and variants selected from GenBank, dbEST, and RefSeq. Hybridization controls were spiked into the cRNA samples to monitor and troubleshoot the hybridization process. Probes for housekeeping genes were used to assess sample integrity. Hybridization, washing, staining, and scanning were performed using Affymetrix GeneChip system instruments. Affymetrix GeneAtlas instrument control software version 1.0.5.267 was used to analyze microarray image data and to compute intensity values. Affymetrix .CEL files containing raw, probe-level signal intensities were analyzed using Partek Genomics Suite version 6.6.12.0713 (Partek). Robust multichip averaging (RMA) was used for background correction, quantile normalization, and probe set summarization with median polish. Statistical differences were calculated by two-way analysis of variance (ANOVA) with a false discovery rate (FDR) of 0.05. Finding common transcripts between compared groups (Fig. 10A) was done using RStudio (version 1.0.153) running R (version 3.4.1).

### Western blot analysis.

Suit2 cells (1.0 × 10^6^ cells) were seeded in 6-well plates in DMEM culture medium containing 10% FBS. When cells became confluent, Suit2 cells were treated or mock treated for 4 or 24 h with compounds (3 μM paclitaxel or 3 μM ruxolitinib) and then treated or mock treated with 5,000 U/ml IFN-α (catalog number 407-294; Millipore) for 4 h. In another set of experiments, confluent Suit2 cells were pretreated for 24 h with medium, 500 nM paclitaxel, or 500 nM colchicine. Next, cells were washed with PBS and infected or mock infected with 500 μl of VSV-ΔM51 at different MOIs for 1 h at 37°C in culture medium without FBS. Virus and media were then removed, and 1 ml of medium (5% FBS) was added. Cells were harvested at 1, 8, and 24 h p.i. and lysed in lysis buffer containing 1 M Tris-HCl (pH 6.8), 10% glycerol, 2% SDS, 5% beta-mercaptoethanol, and 0.02% (wt/vol) bromophenol blue. Total protein was separated by electrophoresis on SDS-PAGE gels and electroblotted onto polyvinylidene difluoride (PVDF) membranes. Membranes were blocked using 5% nonfat powdered milk in Tris-buffered saline–Tween 20 (TBS-T) (0.5 M NaCl, 20 mM Tris [pH 7.5], 0.1% Tween 20). Membranes were incubated in TBS-T with 5% bovine serum albumin (BSA) or milk with 0.02% sodium azide and a 1:5,000 dilution of rabbit polyclonal anti-VSV antibodies (raised against VSV virions), a 1:500 dilution of rabbit anti-phospho-STAT1 (catalog number 7649S, clone Y701; Cell Signaling), a 1:500 dilution of rabbit anti-STAT1 (catalog number 14994T, clone D1K9Y; Cell Signaling), a 1:1,000 dilution of mouse anti-STAT2 (catalog number MAB1666; R&D Systems), a 1:500 dilution of rabbit anti-phospho-STAT2 (catalog number MAB2890, clone Y689; R&D Systems), or a 1:1,000 dilution of rabbit anti-cyclin B1 (clone D5C10; Cell Signaling). A 1:2,000 dilution of goat anti-rabbit peroxidase-conjugated secondary antibodies (Jackson-ImmunoResearch) was used. The Amersham ECL Western blotting detection kit (GE Healthcare) was used for detection. To verify total protein in each loaded sample, membranes were reprobed with mouse antiactin antibody (catalog number MA5-15739; Thermo Fisher) or rabbit anti-glyceraldehyde-3-phosphate dehydrogenase (GAPDH) antibody (catalog number sc-25778; Santa Cruz) or stained with Coomassie blue R-250.

### ELISA.

Production of IFNs in supernatants of cell cultures was analyzed using the following commercial enzyme-linked immunosorbent assay (ELISA) kits: the VeriKine human IFN-α multisubtype ELISA kit (catalog number 41105; PBL Assay Science), the VeriKine human IFN beta ELISA kit (catalog number 41410; PBL Assay Science), and the human IL-28A ELISA kit (catalog number EHIL28A; Thermo Fisher Scientific).

### Confocal microscopy.

Suit2 cells were seeded in Lab-Tek II chambered cover glass with a no. 1.5 borosilicate glass bottom (Thermo Fisher Scientific) at 50% confluence and treated or mock treated with 500 nM paclitaxel, 500 nM docetaxel, 500 nM colchicine, 3 μM aphidicolin, or 2 mM thymidine for 24 h. Cells were washed in PBS, incubated for 10 min at 37°C with CellMask Deep Red plasma membrane stain (catalog number C10046; Life Technologies), and diluted 1:1,000 in the medium. Cells were then washed 3 times with PBS, incubated for 10 min at 37°C with Hoechst-33342 (Thermo Fisher Scientific), and diluted 1:1,000 in PBS. Next, cells were washed 3 more times with PBS and fixed in 3.75% formaldehyde for 5 min. After 3 additional washes in PBS, cells were dried and imaged using a confocal microscope (Olympus FluoView1000), using filters for Hoechst-33342 (blue) and Alexa Fluor 594 (red).

### Statistical analysis.

All statistical analyses were performed using GraphPad Prism 7.0a software. Tests used are indicated in the legends of the figures.

## Supplementary Material

Supplemental file 1

Supplemental file 2

Supplemental file 3

Supplemental file 4
